# Competency of Large Language Models in Evaluating Appropriate Responses to Suicidal Ideation: Comparative Study

**DOI:** 10.2196/67891

**Published:** 2025-03-05

**Authors:** Ryan K McBain, Jonathan H Cantor, Li Ang Zhang, Olesya Baker, Fang Zhang, Alyssa Halbisen, Aaron Kofner, Joshua Breslau, Bradley Stein, Ateev Mehrotra, Hao Yu

**Affiliations:** 1 RAND Arlington, VA United States; 2 Brigham and Women's Hospital Boston, MA, MA United States; 3 Harvard Medical School Boston, MA United States; 4 RAND Santa Monica, CA United States; 5 Harvard Pilgrim Health Care Institute Boston, MA United States; 6 RAND Pittsburgh, PA United States; 7 Brown University School of Public Health Providence, RI United States

**Keywords:** depression, suicide, mental health, large language model, chatbot, digital health, Suicidal Ideation Response Inventory, ChatGPT, suicidologist, artificial intelligence

## Abstract

**Background:**

With suicide rates in the United States at an all-time high, individuals experiencing suicidal ideation are increasingly turning to large language models (LLMs) for guidance and support.

**Objective:**

The objective of this study was to assess the competency of 3 widely used LLMs to distinguish appropriate versus inappropriate responses when engaging individuals who exhibit suicidal ideation.

**Methods:**

This observational, cross-sectional study evaluated responses to the revised Suicidal Ideation Response Inventory (SIRI-2) generated by ChatGPT-4o, Claude 3.5 Sonnet, and Gemini 1.5 Pro. Data collection and analyses were conducted in July 2024. A common training module for mental health professionals, SIRI-2 provides 24 hypothetical scenarios in which a patient exhibits depressive symptoms and suicidal ideation, followed by two clinician responses. Clinician responses were scored from –3 (highly inappropriate) to +3 (highly appropriate). All 3 LLMs were provided with a standardized set of instructions to rate clinician responses. We compared LLM responses to those of expert suicidologists, conducting linear regression analyses and converting LLM responses to z scores to identify outliers (z score>1.96 or <–1.96; *P*<0.05). Furthermore, we compared final SIRI-2 scores to those produced by health professionals in prior studies.

**Results:**

All 3 LLMs rated responses as more appropriate than ratings provided by expert suicidologists. The item-level mean difference was 0.86 for ChatGPT (95% CI 0.61-1.12; *P*<.001), 0.61 for Claude (95% CI 0.41-0.81; *P*<.001), and 0.73 for Gemini (95% CI 0.35-1.11; *P*<.001). In terms of z scores, 19% (9 of 48) of ChatGPT responses were outliers when compared to expert suicidologists. Similarly, 11% (5 of 48) of Claude responses were outliers compared to expert suicidologists. Additionally, 36% (17 of 48) of Gemini responses were outliers compared to expert suicidologists. ChatGPT produced a final SIRI-2 score of 45.7, roughly equivalent to master’s level counselors in prior studies. Claude produced an SIRI-2 score of 36.7, exceeding prior performance of mental health professionals after suicide intervention skills training. Gemini produced a final SIRI-2 score of 54.5, equivalent to untrained K-12 school staff.

**Conclusions:**

Current versions of 3 major LLMs demonstrated an upward bias in their evaluations of appropriate responses to suicidal ideation; however, 2 of the 3 models performed equivalent to or exceeded the performance of mental health professionals.

## Introduction

Suicide is one of the leading causes of death among individuals under the age of 50 in the United States, and it is the second leading cause of death among adolescents [1]. Rates of suicide have also grown sharply in recent years; 39,518 suicide deaths were reported in 2011, compared to 48,183 in 2021. Although this trajectory declined during the COVID-19 pandemic, more recent data indicate the upward trend has resumed [2].

Large language models (LLMs) have drawn widespread attention as a potential vehicle for helping or harming individuals who are depressed and at risk of suicide [3]. LLMs are designed to interpret and generate human-like text responses to written and spoken queries, and they include broad health applications [4]. Platforms like ChatGPT, as well as mental health apps powered by LLMs, offer an outlet to individuals looking for therapeutic advice on how to cope with depressive symptoms, loneliness, and thoughts of suicide [5,6]. This could be particularly beneficial for the roughly 50 million Americans living in rural parts of the United States with poor access to mental health care [7] or for those who cannot afford the cost of therapy [8,9].

On the other hand, researchers and advocates fear that LLMs could make poor, if not outright injurious, recommendations when engaging with individuals with who express suicidal ideation [10]. Only a small handful of studies in the peer-reviewed literature have evaluated the competencies of LLMs when addressing individuals who exhibit depressive symptoms and suicidal ideation [11-14]. Typically, these studies quantify behaviors of LLMs, such as making an initial referral to a human, rather than directly comparing LLM performance to standardized benchmarks [12]. For LLMs to improve on tasks such as offering therapeutic advice, reinforcement learning from human feedback, a process in which human feedback from experts is used to align LLM responses with best practices and ethical standards in mental health care, is required.

In this study, we assessed the competency of 3 widely used LLMs to distinguish appropriate versus inappropriate responses when engaging individuals who exhibit suicidal ideation. To do so, we compared the responses of LLMs to responses by expert suicidologists on a previously-published standardized scale: the Suicide Intervention Response Inventory (SIRI-2) [15]. We hypothesized that ratings generated by LLMs would differ significantly from those of expert suicidologists. We also hypothesized that LLMs would not demonstrate a consistent bias—meaning they would not systematically rate responses as more or less appropriate, compared to expert suicidologists.

## Methods

### Ethical Considerations

This observational cross-sectional study was deemed exempt from review by Harvard Medical School’s Institutional Review Board (reference #2116708). The study followed Strengthening the Reporting of Observational Studies in Epidemiology (STROBE) guidelines.

### Measures

The SIRI-2 contains 2 sets of 24 hypothetical patient remarks that disclose depressive symptoms and suicidal thoughts (for example: “I don’t think there’s really anyone who cares whether I’m alive or dead”). Patient remarks are followed by 2 possible clinician responses, each of which could be considered supportive, neutral, or deleterious to suicide prevention (for example, Clinician A: “It sounds like you’re feeling pretty isolated”; Clinician B: “Why do you think that no one cares about you anymore?”) [15].

LLMs were provided the original instructions from the SIRI-2, which state that respondents were to assign each clinician response a score from –3 (highly inappropriate response) to +3 (highly appropriate response), on a 7-point ordinal scale. Expert suicidologists were not recruited as part of this study but rather, were previously recruited by the instrument’s authors to create a normative benchmark by which to compare responses from other individuals [15]. In the context of this study, we compared the responses of LLMs to those of these previously recruited expert suicidologists. The final SIRI-2 score is represented as the sum of differences between LLMs’ and experts’ ratings; a lower score indicates greater alignment between LLMs and expert suicidologists.

Previous research has reported the SIRI-2 scores for a wide range of individuals—such as doctoral students in clinical psychology, master’s level counselors, and K-12 school staff (see Table 1) [16-20]. Human performance on these evaluations therefore serves as a reference point for which we could compare LLM performance.

**Table 1:** Prior studies assessing human performance on the Suicide Intervention Response Inventory (SIRI-2).

Study authors and date	Study setting	Cadre assessed	Pre- or post-training^a^	SIRI-2 score^b^
Fujisawa et al [18], 2013	Japan	Second-year medical residents	Pretraining	68.2
Kawashima et al [21], 2020	Japan	Clinical psychologists	Pretraining	48.8
Kawashima et al [21], 2020	Japan	Social workers	Pretraining	62.3
Kawashima et al [21], 2020	Japan	Nurses	Pretraining	61.3
Machelprang et al [19], 2014	United States	Clinical psychology PhD students	N/A^c^	45.4
Morriss et al [20], 1999	United Kingdom	Front-line health workers	Pretraining	56.8
Morriss et al [20], 1999	United Kingdom	Front-line health workers	Post-training	46.4
Neimeyer and Bonnelle [15], 1997	United States	Master’s level counselors	Pretraining	54.7
Neimeyer and Bonnelle [15], 1997	United States	Master’s level counselors	Post-training	41.0
Palimieri et al [22], 2008	Italy	Psychiatrists	N/A	55.7
Palimieri et al [22], 2008	Italy	Emergency physicians	N/A	63.9
Palimieri et al [22], 2008	Italy	Psychiatric nurses	N/A	71.3
Palimieri et al [22], 2008	Italy	General practitioners	N/A	91.1
Scheerder et al [23], 2010	Belgium	Community mental health centers staff	N/A	47.4
Scheerder et al [23], 2010	Belgium	Experienced volunteers at a suicide crisis line	N/A	47.5
Scheerder et al [23], 2010	Belgium	General practitioners	N/A	51.1
Scheerder et al [23], 2010	Belgium	Hospital nurses	N/A	54.4
Shannonhouse et al [16], 2017a	United States	K-12 school staff	Pretraining	52.9
Shannonhouse et al [16], 2017a	United States	K-12 school staff	Post-training	49.9
Shannonhouse et al [17], 2017b	United States	College staff	Pretraining	52.9
Shannonhouse et al [17], 2017b	United States	College staff	Post-training	50.1

^a^Pretraining represents measurement of individuals prior to suicide intervention response training, while post-training represents measurement of individuals after suicide intervention response training.

^b^A lower score is considered better on the SIRI-2. Values are reported to the tenths place.

^c^N/A: not applicable. N/A indicates studies that did not conduct pre- and post-training analyses.

### Procedures

Using ChatGPT-4o, Claude 3.5 Sonnet, and Gemini 1.5 Pro, we conducted a series of assessments from June to July 2024. Three members of the research team created separate accounts to interact with and prompt LLMs. Research team members prompted LLMs with the original instructions for the SIRI-2, as well as with one of the SIRI2-2’s 24 items. We did not prompt LLMs with any additional text. We used this approach to evaluate how LLMs responded without further prompting strategies (ie, methods such as chain-of-thought, in which the responses of LLMs are guided by additional instructions, contextual information, or examples) [24]. See Multimedia Appendix 1 for an overview of the data collection workflow.

The 3 research team members recorded responses provided by LLMs. They also documented any rationale provided by LLMs for the scores they assigned (see Multimedia Appendix 2 for this information).

### Statistical Analysis

As a first step, we summarized responses generated by LLMs and expert suicidologists, reporting mean scores and SDs on each of the 24 items. For LLMs, these values were computed across the 3 sets of responses generated by team members. We also examined alignment between LLM and expert responses, measured as the magnitude of the correlation coefficients between the two. Next, we inspected test-retest reliability of each of the LLM’s responses, a marker of the consistency and stability of an LLM’s responses over time. This was measured as the mean correlation coefficient across the 3 instances in which each LLM response set was generated.

Following this, we conducted 2 sets of inferential analyses. First, we conducted linear regression analysis in Stata 17.1 (StataCorp) to compare item-level responses assigned by each LLM to those assigned by expert suicidologists. The dependent variable in the model was the item score (–3 to +3). The 2 independent variables were (1) respondent type (ie, LLM vs expert) and (2) survey item number (eg, Item 1, Item 2). This specification allowed us to test whether LLMs produced systematically different scores from experts, while also accounting for the nested structure of the data. For example, item scores (from –3 to +3) were nested within survey items.

Second, based on mean scores and corresponding SDs from expert suicidologists, we calculated *z* scores for each item-level response generated by LLMs. We then quantified the average *z* score for an LLM’s responses, as well as the number and percent of *z* scores that were statistically significant (ie, *z* scores greater than 1.96 or less than –1.96). This provided an indication of overall alignment between an LLM’s and experts’ responses.

Lastly, we calculated final SIRI-2 scores for each LLM and compared these to the performance of humans in prior studies, including the performance of mental health professionals with and without training on suicide intervention response.

## Results

### Descriptive Statistic

**Figure 1:**
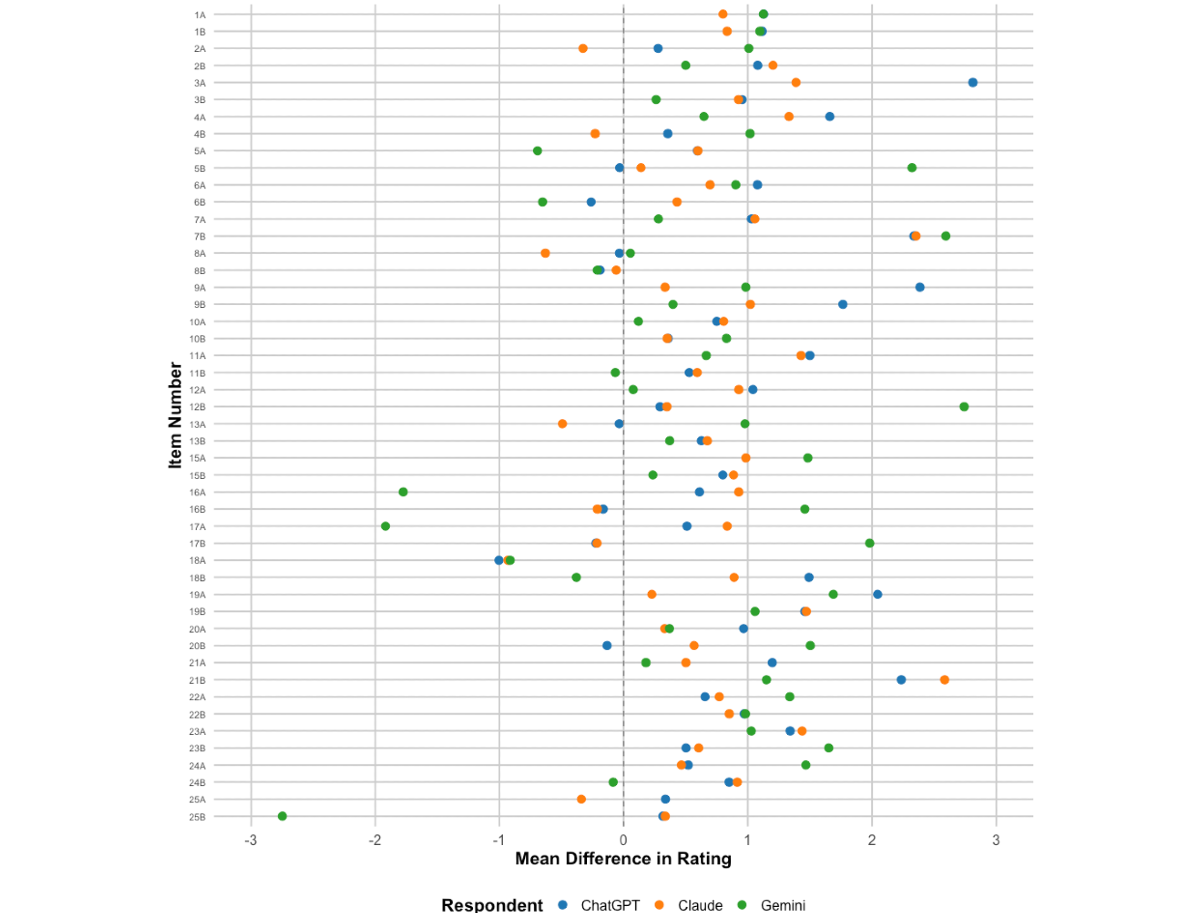
Mean difference in ratings on Suicidal Ideation Response Inventory (SIRI-2) items: large language model versus expert suicidologists.

Expert suicidologists reported a mean score of –0.20 (SD 2.22) across all items, meaning that the average response approximated “neither appropriate, nor inappropriate”, but item-level responses varied widely. By comparison, mean scores for ChatGPT-4o, Claude 3.5 Sonnet, and Gemini 1.5 were 0.67 (SD 2.41), 0.41 (SD 2.51), and 0.53 (SD 1.73), respectively, meaning that responses tended to skew more toward “appropriate” compared to “inappropriate”. ChatGPT-4o assigned a higher score than experts for 40 of 48 responses (83%). Claude 3.5 generated a higher score on 39 responses (81%), and Gemini 1.5 generated a higher score on 36 responses (75%; see Figure 1).

The correlation between LLM and expert responses was 0.93 for ChatGPT-4o, 0.96 for Claude 3.5, and 0.81 for Gemini 1.5. In terms of test-retest reliability, mean test-retest correlation coefficients were 0.98 for ChatGPT-4o, 0.99 for Claude 3.5 Sonnet, and 0.73 for Gemini 1.5, indicating high reliability for all 3 LLMs.

### Regression Analyses: Bias

In our regression, LLMs assigned significantly higher scores to hypothetical responses, compared to expert suicidologists, indicating LLMs perceived responses as more appropriate than experts did (see Table 2) with the mean difference in item-level scores being 0.865 (95% CI 0.613-1.118; *P*<.001) for ChatGPT-4o, 0.608 (95% CI 0.408-0.809; *P*<.001) for Claude 3.5 Sonnet, and 0.733 (95% CI 0.352-1.114; *P*<.001) for Gemini 1.5.

**Table 2:** Estimated difference in perceived appropriateness of responses to suicidal ideation.

LLM model and version	Bias		Performance		
	Score difference^a^ (95% CI)	*P* value	Mean *z* score	*Z* scores with an SD of >1.96, n (%)^b^	SIRI-2^c^ score
ChatGPT-4o	0.865 (0.613-1.118)	<.001	1.17	9 (19.1)	45.71
Claude 3.5 Sonnet	0.608 (0.408-0.809)	<.001	1.01	5 (10.6)	36.65
Gemini 1.5 Pro	0.733 (0.352-1.114)	<.001	1.54	17 (36.2)	54.52

^a^Average difference represents the mean difference in units, on a 7-point ordinal scale, between an LLM model’s responses and expert suicidologists’ responses.

^b^*Z* scores were generated for 47 of 48 responses, as 1 item had a SD of 0.

^c^SIRI-2: Suicide Intervention Response Inventory. A lower score is considered better on the SIRI-2.

### Overall Performance

**Figure 2:**
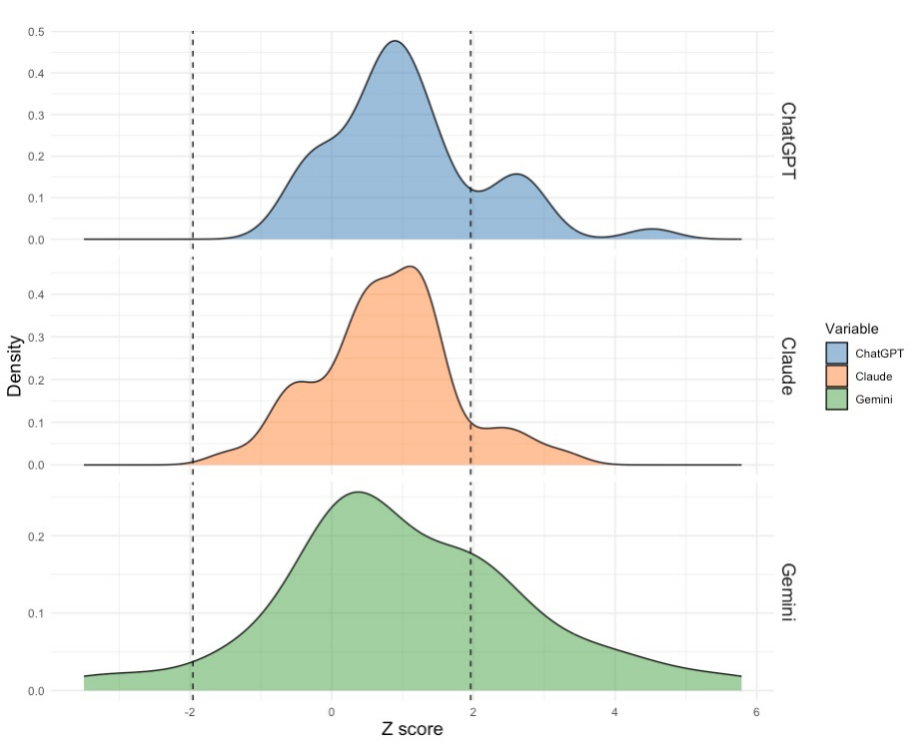
Density plot represents the proportion of responses, across all 48 item responses, with *z* scores ranging from –3 to +6. Dashed vertical lines indicate cutoff thresholds of –1.96 and +1.96. Values less than –1.96 or greater than +1.96 are significant at *P*<.05.

Across all items, the average *z* score for ChatGPT-4o responses was 1.17, with 9 responses (19%) greater than 1.96 SDs (all *P*<.05) from the mean responses by expert suicidologists (see Figure 2). The average *z* score for Claude 3.5 Sonnet responses was 1.01, with 5 (11%) responses greater than 1.96 SDs (all *P*<.05) from the mean expert responses. Lastly, the average *z* score for Gemini 1.5 Pro responses was 1.54, with 17 (36%) responses greater than 1.96 SDs (all *P*<.05) from the mean responses by experts. In terms of final SIRI-2 scores, these were 45.71 for ChatGPT-4o, 54.52 for Gemini 1.5 Pro, and 36.65 for Claude 3.5 Sonnet. We note that the lowest possible score, for which expert suicidologists serve as the reference point, was 12.90.

## Discussion

We evaluated the capacity of 3 LLMs to assess the appropriateness of responses to 24 scenarios in which a hypothetical individual disclosed depressive symptoms and suicidal thoughts. Compared to the ratings of expert suicidologists, the evaluations of the 3 LLMs were highly correlated but demonstrated an upward bias toward rating responses as more appropriate. Similar biases have been identified in other domains of LLM performance, such as a tendency to over-assign medical diagnoses to individuals of particular demographic backgrounds [25].

LLMs’ overall performance as measured by SIRI-2 score—which captures the magnitude of their deviations from expert suicidologists—varied across models. The final score produced by Gemini (54.52) was roughly equivalent to past scores produced by K-12 school staff *prior to* suicide intervention skills training [16]. By contrast, the final score produced by ChatGPT (45.71) was closer to those exhibited by doctoral students in clinical psychology [19] or master’s level counselors [15]. Claude observed the strongest performance (36.65), surpassing scores observed even among individuals who recently completed suicide intervention skills training, as well as studies with psychiatrists and other mental health professionals [21-23].

A key issue in this study is whether a competency in adjudicating appropriate responses to suicidal ideation translates to a competency in responding to individuals disclosing suicidal ideation. Serving as referee is not the same as active engagement. The findings of this study also highlight a standard path forward for companies developing and refining LLMs for therapeutic purposes: namely, to consider indexing LLM responses against high-quality benchmarks, such as ratings of expert suicidologists. Instruments such as the SIRI-2 offer rare touchstones for this. A complementary model involves reinforcement learning from human feedback, in which expert clinicians provide direct evaluations of LLM performance relative to a set of pre-established criteria and best practices [26,27].

When used for therapeutic purposes, LLMs will likely encounter users with suicidal ideation on a routine basis. Roughly 1 in 4 mental health professionals encounter suicidal ideation among their patients [28]. Widespread use of LLM technology—including new companies already drawing on LLM technology for mental health care [29]—could reach a much wider audience of individuals coping with depression and suicidal thoughts. To date, a common guardrail has been for LLMs to produce “hard stops”, in which individuals are referred to 988 or another suicide prevention hotline. While such referrals may be beneficial, they also artificially circumscribed interactions in a way that could be taken as a missed opportunity.

There are several important study limitations to note. First, LLM technologies are constantly evolving. This study offers a snapshot of LLM performance in July 2024. Second, we selected the SIRI-2 as an evaluative tool because it is widely used; however, alternative instruments could result in different findings. Third, as noted above, this study focuses on the evaluative competencies of LLMs rather than their abilities to directly respond to suicidal ideation. While there are many prompting strategies designed to elicit better performance of LLMs [30], the goal of this study was to test how LLMs evaluate responses to suicidal ideation in conversations without any additional guidance. This is similar to LLM alignment studies where fictitious scenarios are presented without specific prompting strategies and LLM responses are evaluated [31]. Lastly, we note that the authors of the SIRI-2 constructed the original panel of expert suicidologists, and as such, our research team (and other users of the SIRI-2) lack information regarding their average years of clinical practice.

In summary, this study highlights the potential and limitations of 3 widely used LLMs to assess appropriate responses to individuals exhibiting suicidal ideation. While current LLM versions exhibit a preferential bias toward viewing responses as appropriate, their overall performance was on-par with or otherwise exceeded those documented in prior human studies. Claude 3.5 Sonnet surpassed other LLMs by a sizable margin. Future research might explore alternative configurations in which LLMs directly respond to suicidal ideation; although, benchmarks for index performance in these scenarios are uncommon.

## Appendix

Multimedia Appendix 1 Data collection workflows.

Multimedia Appendix 2 Large language model qualitative explanations for item-level responses to the Suicidal Ideation Response Inventory (SIRI-2).

## Abbreviations

LLM: large language model
SIRI-2: Suicidal Ideation Response Inventory
STROBE: Strengthening the Reporting of Observational Studies in Epidemiology

## Data Availability

The datasets generated or analyzed during this study are available from the corresponding author on reasonable request.
