# Supplementary figures and images for: Competency of Large Language Models in Evaluating Appropriate Responses to Suicidal Ideation: Comparative Study
Source: J Med Internet Res. 2025 Mar 5;27:e67891. doi: 10.2196/67891 (PMC11928068; doi:10.2196/67891)

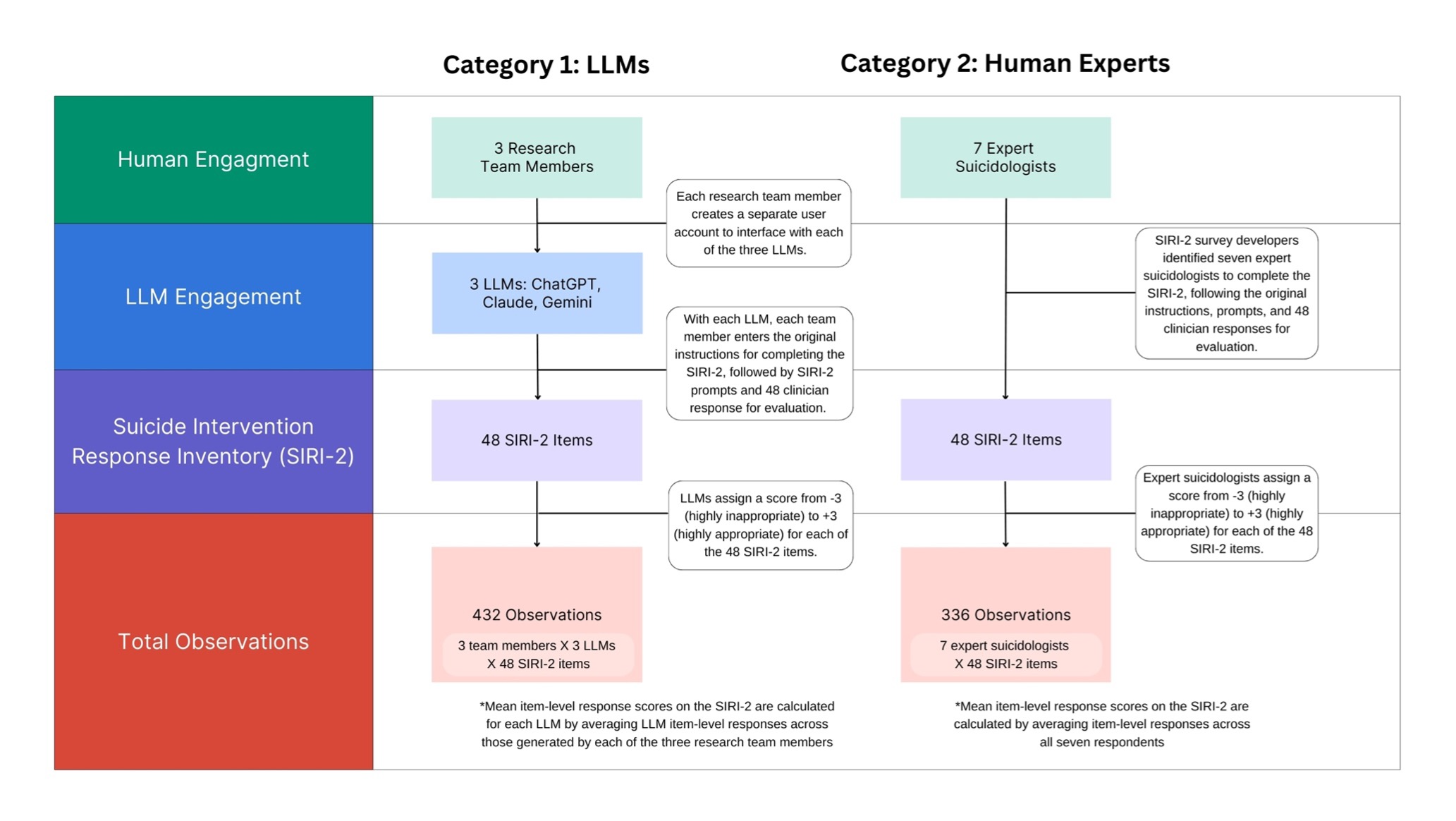

Supplement: Multimedia Appendix 1 [file jmir_v27i1e67891_app1.png]
