# Supplementary material for: Competency of Large Language Models in Evaluating Appropriate Responses to Suicidal Ideation: Comparative Study
Source: J Med Internet Res. 2025 Mar 5;27:e67891. doi: 10.2196/67891 (PMC11928068; doi:10.2196/67891)
Supplement: Multimedia Appendix 2 [file jmir_v27i1e67891_app2.docx]

**Online Supplement**

**Appendix Table 1. LLM Qualitative Explanations for Item-Level Responses to the SIRI-2.**

| **Item** | **Expert**  **Score** | **ChatGPT-4o** | | **Claude 3.5 Sonnet** | | **Gemini 1.5 Pro** | |
| --- | --- | --- | --- | --- | --- | --- | --- |
|  |  | **Mean Score** | **Offered Explanation** | **Mean Score** | **Offered Explanation** | **Mean Score** | **Offered Explanation** |
| 1A | -2.71 | -1.67 | This response might come across as dismissive or minimizing the client's expression of suicidal thoughts by suggesting that there is something else bothering them. It doesn't directly address the seriousness of the suicidal ideation, which is crucial in such a sensitive situation. | -2.00 | This response is inappropriate. While it attempts to explore the underlying issues, it comes across as dismissive of the client's stated suicidal feelings. The phrasing "You say you're suicidal, but..." implies doubt about the seriousness of the client's statement, which could be harmful when dealing with someone expressing suicidal thoughts. | -1.67 | This might seem like a way to get to the root cause, but it dismisses the immediate urgency of the suicidal feelings. It can also feel dismissive or minimizing to the client. |
| 1B | 1.86 | 3.00 | This response directly addresses the client's statement about feeling suicidal, inviting them to elaborate on their feelings. It shows empathy and a willingness to listen, which is crucial in a crisis situation. | 2.67 | This response is highly appropriate. It directly acknowledges the client's statement about suicidal feelings and invites them to elaborate. This approach shows that the helper is listening, taking the client's feelings seriously, and encouraging them to share more, which is crucial in a situation involving suicidal ideation. | 3.00 | It shows empathy and opens the door for the client to elaborate on their thoughts and feelings without judgment. |
| 2A | -2.71 | -2.33 | This response attempts to reassure the client but might come across as dismissive of their very real and immediate feelings of distress and hopelessness. It lacks empathy and does not acknowledge the client's current emotional state. | -3.00 | This response is highly inappropriate. It dismisses the client's very real concerns and emotions with a platitude. Telling someone who is dealing with serious health issues and the loss of a spouse to "not worry" and that "everything will be alright" minimizes their experience and could make them feel misunderstood or invalidated. | -1.67 | Try not to worry" minimizes the client's feelings and dismisses their concern; "Everything will be alright" offers false reassurance and ignores the client's current situation. |
| 2B | 1.86 | 3.00 | This response shows empathy by acknowledging the client's feelings of loneliness and fear. It validates their emotions and opens the door for further discussion about their experiences and feelings. | 3.00 | This response is highly appropriate. It demonstrates empathy and understanding of the client's emotional state. By reflecting back the likely feelings of loneliness and fear, the helper shows that they are listening and validates the client's emotions. This approach encourages further discussion and helps build rapport. | 2.33 | Validates the client's feelings of loneliness and fear; Opens the door for them to explore their emotions further. |
| 3A | -2.14 | 0.67 | This response emphasizes the helper's professional qualifications, which might provide some reassurance to the client. However, it may come across as somewhat detached and does not directly address the client's feelings of fear and shame about their thoughts. | -0.67 | While this response attempts to create a safe space for the client to share, it comes across as somewhat dismissive of the client's feelings. The emphasis on being a "professional" and "objective" might unintentionally create distance and make the client feel that their emotions are being analyzed rather than understood. However, it's not entirely inappropriate as it does encourage openness | 1.67 | While offering a safe space to share is positive, "professional" and "objective" might sound distant and impersonal. |
| 3B | 2.14 | 3.00 | This response acknowledges the client's fear and the intensity of their thoughts. It validates their feelings and shows empathy by understanding why the client might be reluctant to share their thoughts. | 3.00 | This response is highly appropriate. It demonstrates empathy and insight into the client's emotional state. By reflecting back the client's feelings about their thoughts (that they are frightening and potentially shocking to others), the helper validates the client's experience without pushing them to disclose before they're ready. This approach shows understanding and may help the client feel more comfortable opening up further. | 2.33 | Accurately reflects the client's fear of judgment; Uses empathy and avoids judgmental language; Encourages exploration of the thoughts without pressuring disclosure. |
| 4A | 1.29 | 3.00 | This response validates the client's feelings and acknowledges the depth of their suffering. It shows empathy and understanding, making it easier for the client to feel heard and supported. | 2.67 | This response is highly appropriate. It demonstrates deep empathy and understanding of the client's emotional state without judging or dismissing their actions. By reflecting back the connection between the client's emotional pain and their self-harming behavior, the helper shows that they are listening and validates the client's experience. This approach can help build trust and encourage further discussion about healthier coping mechanisms. | 2.00 | This response shows empathy by acknowledging the client's pain and validates their experience. It avoids judgment and focuses on understanding the client's coping mechanism. |
| 4B | -2.71 | -2.33 | This response, while well-intentioned, might come across as dismissive and invalidating. It shifts the focus to the client's age and potential future, which can make the client feel misunderstood and minimize their current pain and experiences. | -3.00 | This response is highly inappropriate. It dismisses the client's feelings and experiences by using platitudes ("you have so much to live for") and expresses shock or judgment about their thoughts. This approach could make the client feel misunderstood, invalidated, and less likely to open up further. Additionally, it mistakenly equates self-harm with a suicide attempt, which may not be accurate and could further alienate the client. | -1.67 | This response minimizes the client's pain by focusing on age and minimizes suicidal ideation. It can be dismissive and may push the client away from seeking help. |
| 5A | 2.43 | 3.00 | This response acknowledges the client's skepticism and focuses on understanding the client's feelings. It doesn't take the client's challenge personally and keeps the conversation centered on the client's concerns. | 3.00 | This response is highly appropriate. It demonstrates active listening and effectively reflects the underlying concern in the client's confrontational questions. By reframing the client's statements as a question about understanding, the helper avoids becoming defensive and keeps the focus on the client's feelings and experiences. This approach can help de-escalate the situation and encourage further dialogue. | 1.67 | This response shows empathy and actively listens to the client's concerns. It acknowledges the client's doubt about the therapist's ability to understand and opens the door for a discussion about building trust. |
| 5B | -2.71 | -2.67 | This response is confrontational and shifts the focus away from the client to the helper. It dismisses the client's feelings and may create an adversarial dynamic, making the client feel invalidated and less likely to open up. | -2.67 | This response is confrontational and defensive, which could escalate tension.  It shifts the focus away from the person at-risk and onto the helper's experiences.  It invalidates the person's feelings by comparing experiences.  The phrase "You're not even giving me a chance" sounds accusatory and may make the person feel judged.  Sharing personal information about having a "rough life" is unprofessional and unhelpful in this context. | -0.33 | While it attempts to connect with the client through shared experience, it can be seen as dismissive of the client's unique situation. The focus shifts to the therapist's experiences, taking the spotlight away from the client's struggles. |
| 6A | -2.00 | -1.00 | This response attempts to redirect the person's thoughts to a positive perspective but may come across as dismissive of their current feelings of worthlessness and grief. It may not fully address the depth of the person's pain. | -1.33 | This response is marginally inappropriate. While it attempts to provide perspective by invoking the memory of Emma, it does so in a way that could be perceived as guilt-inducing or dismissive of the client's current feelings. The use of "But try to think..." suggests the client isn't already considering these factors, which might not be true. Additionally, presuming what Emma would want might not be helpful if the client is struggling with grief and loneliness. | -1.00 | While attempting to motivate the client by considering Emma's wishes, it can feel dismissive of the client's current grief and struggles. Assuming what Emma would want puts pressure on the client and may not be helpful. |
| 6B | 2.57 | 2.33 | This response shows empathy and understanding of the client's profound loss. It acknowledges the client's ongoing pain and invites them to explore recent events that might have exacerbated their feelings. However, the phrase "make you think that dying is the only way out" could be seen as somewhat leading, so it's not a perfect response but still more appropriate and empathetic than Helper A's response. | 3.00 | This response is highly appropriate. It acknowledges the profound impact of Emma's death on the client's life while also recognizing that something recent may have exacerbated the situation. By validating the client's feelings of loss and then gently probing for more recent triggers, the helper shows empathy and encourages the client to explore their current state more deeply. This approach can help identify immediate concerns that might be addressable, while still respecting the long-term grief the client is experiencing. | 2.00 | This response shows empathy by acknowledging the loss and the impact it had on the client's life. It gently explores recent events that might be contributing to the suicidal thoughts, opening the door for problem-solving and focusing on the present situation. |
| 7A | 2.00 | 3.00 | This response acknowledges the difficulty the client is having in expressing themselves. It shows empathy and understanding, creating a supportive environment for the client to open up. | 3.00 | This response is highly appropriate. It demonstrates empathy and understanding of the client's emotional state. By acknowledging the difficulty the client is experiencing in expressing their thoughts and feelings, the helper validates the client's struggle and creates a supportive environment. This approach can help the client feel understood and may encourage them to continue when they feel ready. | 2.33 | This response remains the most appropriate (+3) for the following reasons: Empathy: It acknowledges the client's difficulty and validates their emotional state. Safety: It creates a safe space by implying the client doesn't have to rush into details. Opens the Door: It encourages the client to share when they're ready, without pressure. |
| 7B | -1.29 | 1.00 | This response encourages the client to continue talking and indicates a willingness to listen. However, it may feel a bit more pressuring for the client who is already struggling to express themselves, as it doesn’t directly acknowledge the difficulty they’re experiencing. | 1.00 | This response is marginally appropriate. While it does express a willingness to listen, which is positive, it might come across as slightly pushy or impatient. The "Go on" could be perceived as pressuring the client to continue speaking when they might need a moment to collect themselves. However, the statement "I'm here to listen to you talk" is supportive and reassuring, which is why this response isn't rated lower. | 1.33 | While still acceptable (+1), Helper B's response is less effective than A for these reasons: Lacks Empathy: It focuses on moving forward rather than acknowledging the initial difficulty. Pressure to Talk: It might feel demanding for a client who's struggling to open up. Even though both responses encourage talking, Helper A's approach is more supportive and creates a more comfortable space for the client to begin sharing. |
| 8A | -2.29 | -2.33 | This response minimizes the client's feelings and dismisses the severity of their suicidal thoughts. It can make the client feel misunderstood and invalidate their emotional experiences. | -3.00 | This response is highly inappropriate. It dismisses the severity of the client's feelings and experiences. By saying "it really isn't so bad after all," the helper minimizes the client's struggles. The question about killing oneself is leading and potentially harmful, as it might make the client feel judged or misunderstood. This approach could discourage the client from further opening up about their suicidal thoughts. | -2.33 | Here's why this response is inappropriate: Minimizes the Problem: It dismisses the seriousness of the client's suicidal thoughts. Dismissive of Feelings: It invalidates the client's emotional state. Pressures the Client: It implies suicide is not a valid reaction to their struggles. |
| 8B | 2.14 | 2.00 | This response acknowledges the benefit of talking about problems and validates the client's decision to seek help. It reinforces the positive step the client took by reaching out and maintains a focus on the seriousness of their feelings. | 2.00 | This response is appropriate. It acknowledges the positive effect of talking through problems and indirectly praises the client for reaching out for help. The helper also recognizes the seriousness of the client's suicidal feelings without being alarmist. However, it could be even better if it didn't assume the client's reasons for contacting the helper, as this might not be entirely accurate. Despite this small issue, the response is generally supportive and encourages continued dialogue. | 2.00 | This response is more appropriate because: Validates the Client: It acknowledges the helpfulness of talking and validates the client's decision to seek help. Normalizes Feelings: It implies that suicidal thoughts can be present during difficult times. Opens for Discussion: It allows for further exploration of the client's feelings without judgment. |
| 9A | -1.29 | 1.00 | This response acknowledges the client's dissatisfaction and offers an apology. However, it might come across as somewhat dismissive and does not directly address the client's emotional state. | -1.00 | This response is marginally inappropriate. While it shows some acknowledgment of the client's feelings, it focuses on the helper's intentions rather than addressing the client's concerns. The apology might come across as defensive, and it doesn't open up a dialogue about what specifically went wrong or how to improve the situation. This approach doesn't effectively address the client's frustration or provide a path forward. | -0.33 | While apologizing can show empathy, in this situation, it deflects responsibility and avoids addressing the client's specific concern. It can come across as dismissive. |
| 9B | 1.29 | 3.00 | This response acknowledges the client's anger and validates their feelings. It opens the door for the client to express their emotions more fully and provides an opportunity to explore the reasons behind their frustration. | 2.33 | This response directly acknowledges the person's emotional state without becoming defensive.  It validates the person's feelings without judgment.  It's a simple observation that could encourage the person to elaborate on their feelings and why they feel the interaction hasn't been helpful.  The response keeps the focus on the person rather than on the helper's intentions or feelings.  It opens the door for further discussion about what went wrong and how to improve the interaction. | 1.67 | This response acknowledges the client's emotional state (anger) and validates their experience. It opens the door for further exploration of what feels worse and how the counselor can be more helpful. |
| 10A | 2.29 | 3.00 | This response acknowledges the client's concern about the helper's ability to understand and assist. It validates the client's feelings and focuses on their need for understanding and support. | 3.00 | This response is highly appropriate. It demonstrates active listening and effectively reframes the client's challenging questions into a concern about understanding and help. This approach avoids getting into personal details about the helper's life experiences, maintains professional boundaries, and keeps the focus on the client's needs and concerns. It opens the door for further discussion about how the helper can be of assistance, without dismissing or minimizing the client's experience. | 2.33 | This response acknowledges the client's underlying concern (feeling understood) and validates the seriousness of their situation. It opens a space for the client to share their feelings without judgment. |
| 10B | -2.43 | -2.00 | This response shares personal experiences, which might seem like an attempt to relate to the client. However, it risks minimizing the client's feelings by implying that their problems can be easily solved with "more realistic solutions." This approach can feel invalidating and dismissive. | -2.00 | This response is inappropriate. While it attempts to relate to the client's experience, it crosses professional boundaries by sharing personal information. Moreover, the second sentence implies that the client's thoughts of suicide are not "realistic," which could be perceived as judgmental or dismissive. This approach risks alienating the client and shifting the focus away from their needs. It may also set up an unhelpful comparison between the helper's and the client's experiences and coping mechanisms. | -1.67 | Disclosing personal experiences of suicidal thoughts is highly inappropriate in this situation. It takes the focus away from the client's experience and can feel invalidating. Additionally, suggesting suicide is not a realistic solution is dismissive and minimizes the client's struggle. |
| 11A | -2.42 | -1.00 | This response introduces a potential gender stereotype, which may not be relevant or helpful in the context of the client's immediate emotional distress. It could divert the conversation away from the client's feelings and onto a broader societal issue. | -1.00 | This response is marginally inappropriate. While it attempts to explore the client's difficulty with expressing emotions, it makes an assumption about gender roles that may not be relevant or helpful. This approach could potentially redirect the conversation away from the client's immediate emotional needs and towards societal expectations, which might not be the most pressing issue at this moment. It also doesn't directly acknowledge the client's pain or validate their emotions. | -1.67 | This response is dismissive of the client's emotions and introduces gender stereotypes. It implies men shouldn't cry, which can be hurtful and unhelpful. |
| 11B | 2.43 | 3.00 | This response acknowledges and validates the client's emotional pain, showing empathy and understanding. It affirms that the client's tears are a natural response to their suffering. | 3.00 | This response is highly appropriate. It demonstrates empathy and validates the client's emotional experience. By acknowledging the depth of the client's hurt and the difficulty of holding back tears, the helper normalizes the client's emotional response and implicitly gives permission for the client to express their feelings openly. This approach is supportive and encourages the client to process their emotions without judgment. | 2.33 | Helper B's response is much more supportive in this scenario. It validates the client's emotions by acknowledging the pain they're experiencing. It allows the client to feel comfortable expressing their tears without judgment. Overall, Helper B's response is more appropriate and shows empathy for the client's emotional state. |
| 12A | 2.00 | 3.00 | This response acknowledges the client's struggle with finding meaning in their suffering. It validates their feelings and provides an empathetic space for further exploration of their emotions. | 3.00 | This response is highly appropriate. It acknowledges the depth of the client's pain and struggle without challenging their beliefs or trying to provide explanations for their suffering. The helper reflects the client's difficulty in finding meaning in their experiences, which validates their feelings and perspective. This approach shows empathy and understanding, and it leaves room for the client to further explore their thoughts and feelings about faith and meaning. | 2.00 | This response validates the client's struggle with faith by acknowledging the hardship they are facing. It avoids religious judgment but opens the door for further exploration of their beliefs. |
| 12B | -3.00 | -2.67 | This response can be very dismissive and potentially hurtful. It assumes the client's belief system and offers a platitude that might not resonate with the client's current feelings of abandonment and anger. It can make the client feel misunderstood and invalidated. | -2.67 | This response dismisses the person's doubts and pain by offering a religious platitude. It imposes a specific religious interpretation on the situation, which may not align with the person's current beliefs or needs. Suggesting that their suffering is a "test" could be perceived as invalidating or even cruel. It doesn't acknowledge the depth of the person's pain or their struggle with faith. This approach could make the person feel misunderstood and potentially increase their feelings of alienation or anger. | -0.33 | While some might find comfort in the idea of "God's plan," this response can be dismissive for those questioning their faith. It offers little comfort and feels impersonal. |
| 13A | -2.57 | -2.67 | This response is dismissive and invalidates the client's feelings. It minimizes the client's experience by implying that their problems aren't serious because they have material wealth and family support. It fails to acknowledge the client's emotional distress and can make them feel misunderstood and unsupported. | -3.00 | This response is highly inappropriate. It dismisses the client's feelings and experiences by implying that their problems aren't serious because of their seemingly good circumstances. This approach minimizes the client's emotional struggle and could make them feel misunderstood or invalidated. Telling someone who is depressed to "focus more on the positive aspects" oversimplifies their situation and doesn't acknowledge that depression can occur regardless of external circumstances. | -1.67 | This response minimizes the client's feelings and dismisses their experience. It can be hurtful and invalidate their struggle with depression. |
| 13B | 2.29 | 3.00 | This response acknowledges that despite external appearances of a good life, the client is experiencing depression. It validates the client's feelings and opens up a space for them to explore their emotions without judgment. | 3.00 | This response is highly appropriate. It acknowledges the paradox between the client's outwardly successful life and their internal emotional state. By reflecting that life seems depressing "even if it's hard to say exactly why," the helper validates the client's feelings without requiring them to justify their emotions. This approach demonstrates understanding of the complex nature of depression and opens the door for further exploration of the client's feelings and experiences. It shows empathy and creates a safe space for the client to discuss their struggles without judgment. | 2.67 | This response validates the client's emotional state by acknowledging the disconnect between their seemingly fortunate situation and their lack of interest. It opens the door for exploring the root of their depression without judgment. |
| 15A | -2.57 | 1.00 | This response acknowledges the prevalence of mental illness and the possibility of treatment, which is helpful. However, it might feel somewhat clinical and less empathetic, potentially making the client feel like just another case rather than an individual in distress. | -1.67 | This response is inappropriate. While it attempts to normalize the client's experience, it does so by immediately labeling their feelings as "mental illness," which could be stigmatizing and may not accurately reflect the client's situation. The use of clinical language like "patients" and "cured" medicalizes the client's experience in a way that might be alienating. Additionally, it doesn't directly address the client's concerns about their thoughts being "dreadful" or "sinful." | -1.00 | This response, while factually accurate about mental illness, uses some potentially triggering language. "Cured" can feel dismissive of the ongoing challenges of mental health. It also focuses on diagnosis over empathy. |
| 15B | 2.14 | 3.00 | This response directly addresses the client's feelings of isolation and validates their experience by normalizing it. It also offers hope by indicating that help is available, which can be very comforting in a crisis. | 3.00 | This response is highly appropriate. It directly and compassionately addresses the client's question, confirming that their experience is not unique. By stating "You're not the only one," it normalizes the client's thoughts without minimizing them. The helper also offers hope by mentioning that help is available and that others have successfully navigated similar crises. This approach validates the client's feelings while providing reassurance and encouragement. | 2.33 | Helper B's response is more supportive and uses less clinical language. It normalizes the client's experience by stating they're not alone in having suicidal thoughts. It offers hope by mentioning others have gotten through similar struggles. |
| 16A | 2.14 | 2.67 | This response shows empathy by acknowledging the client's feelings of loneliness and misery. It also directly addresses the potential risk of suicidal feelings, which is important for assessing the client's safety. | 3.00 | This response is highly appropriate. It demonstrates empathy by reflecting the client's expressed feelings of loneliness and exhaustion. The helper acknowledges the depth of the client's distress and directly, yet gently, inquires about suicidal thoughts. This approach shows that the helper is listening, takes the client's pain seriously, and is willing to discuss difficult topics. It opens the door for the client to talk about suicidal ideation if present, which is crucial for risk assessment and providing appropriate support. | 0.33 | While directly asking about suicide can be helpful in assessing risk, it can also be startling for some clients, especially early in a session. It shows the helper is taking the client's feelings seriously but might be a bit too direct. |
| 16B | -2.86 | -3.00 | This response dismisses the client's feelings and minimizes their distress. It can make the client feel invalidated and misunderstood, which is not helpful in a crisis situation. | -3.00 | This response is highly inappropriate. It dismisses and minimizes the client's expressed feelings of despair. The phrase "Come on now" could be perceived as patronizing, and saying "Things can't be all that bad" invalidates the client's experience. This approach risks making the client feel misunderstood and could discourage them from sharing further or seeking help in the future. It fails to address the potential severity of the situation and does not provide any meaningful support or empathy. | -1.33 | Minimizing the client's feelings ("Things can't be that bad") is dismissive and unhelpful. It invalidates their experience and discourages them from opening up further. |
| 17A | 1.57 | 2.00 | This response offers a practical solution that might make it easier for the client to communicate despite the difficult circumstances. It shows a willingness to adapt to the client's needs. | 2.33 | This response is appropriate. It shows that the helper is attentive to the client's difficulty in communicating and offers a potential solution. By suggesting asking questions, the helper provides a way for the conversation to continue that might be easier for the client in their current situation. This approach demonstrates flexibility and a willingness to adapt to the client's needs. | -0.33 | While directly addressing suicidal thoughts can be important in some cases, here it might be premature. The client hasn't explicitly mentioned suicide, and focusing on it first could overwhelm them. |
| 17B | -1.71 | -2.00 | This response suggests postponing the conversation, which may not be helpful if the client is in immediate distress. It risks missing the opportunity to provide support when the client needs it most. | -2.00 | This response is inappropriate. While it acknowledges the client's difficulty in talking, it suggests ending the current conversation without knowing the urgency of the client's situation. For someone reaching out in crisis, suggesting they call back later could be dismissive and potentially dangerous. It doesn't offer immediate support and might discourage the person from seeking help again. A more appropriate response might combine elements of both, such as offering to ask yes/no questions for easier responses, while also ensuring the client is in a safe place and offering alternatives if talking is truly not possible at the moment. | 0.33 | Helper B's response is more appropriate here. It prioritizes the client's comfort and acknowledges their need for privacy. It offers an opportunity to reschedule when the client feels more secure. |
| 18A | -2.00 | -3.00 | This response is highly inappropriate as it drastically underestimates the gravity of the situation. The client's life is in immediate danger, and this response fails to address the urgency or provide any immediate support. | -3.00 | This response is highly inappropriate. It drastically understates the severity of the situation and fails to address the immediate danger. Using the phrase "somewhat upset" in response to someone threatening suicide with a gun is a severe underreaction that could potentially escalate the situation. | -3.00 | This response is a massive understatement of the situation and minimizes the client's immediate danger. It could escalate the situation. |
| 18B | 1.43 | 3.00 | This response directly addresses the immediate danger and urges the client to put down the gun, prioritizing their safety. It shows a clear understanding of the critical nature of the situation and opens the door for further conversation once the immediate threat is mitigated. | 2.33 | This response directly addresses the immediate danger. It gives a clear, simple instruction that could help de-escalate the situation. The helper shows appropriate concern for the person's safety. It implies a willingness to continue the conversation after the immediate danger is addressed. The tone is direct without being overly authoritative or confrontational. | 1.00 | This is a much more appropriate response. It prioritizes the client's safety by calmly asking them to put down the weapon while opening the door for further communication |
| 19A | -2.29 | -0.33 | This response may come across as impersonal and professional, lacking the emotional depth needed in such a situation. It does not address the client's need for genuine human concern and support. | -2.00 | This response is inappropriate. While it attempts to explain why the helper cares, it comes across as impersonal and suggests that the helper only cares because it's their job, not because they genuinely care about the person. This could make the person at-risk feel even more isolated and reinforce their belief that no one truly cares about them. | -0.67 | While Helper A technically addresses the concern, it feels impersonal and robotic. It focuses on the helper's job rather than building a genuine connection with the client. |
| 19B | 1.57 | 3.00 | This response shows genuine concern for the client's well-being and emphasizes the importance of their life. It acknowledges the severity of the situation and offers a supportive stance, encouraging the client to seek help. | 3.00 | This response is highly appropriate. It directly addresses the person's question with genuine concern and empathy. It validates the seriousness of their situation while also expressing that their life has value. The helper acknowledges the critical nature of the situation and offers support, which can help build trust and open up further dialogue. | 2.67 | Helper B directly addresses the client's feelings of worthlessness and expresses genuine concern. Highlighting the tragic nature of suicide validates the client's struggle while offering support. Mentioning a "critical period" offers hope for improvement. |
| 20A | 2.00 | 3.00 | This response validates the client's feelings of anger and acknowledges the pain caused by the father's lack of support. It shows empathy and understanding, making the client feel heard and supported. | 2.33 | This response is appropriate. The helper acknowledges and validates the person's feelings of anger and hurt. By reflecting the emotion back to the client, the helper shows empathy and understanding. This approach can help the person feel heard and understood, which is crucial in building trust and encouraging further exploration of these feelings. | 2.33 | Helper A validates the client's feelings of anger by acknowledging the cause. This shows empathy and encourages the client to explore their emotions further. |
| 20B | -2.86 | -3.00 | This response dismisses the client's feelings and imposes a moral judgment about how the client should feel. It invalidates the client's emotions and may make them feel misunderstood and further distressed. | -2.33 | This response is inappropriate. It dismisses the person's feelings and experiences, telling them how they "should" feel instead of validating their emotions. This approach can make the person feel misunderstood and judged, potentially damaging the therapeutic relationship. It also assumes that all fathers deserve respect regardless of their actions, which may not be helpful or accurate in this situation. | -1.33 | Helper B dismisses the client's feelings and attempts to impose societal expectations. It's important to allow the client to express their emotions without judgment, even if they are negative towards a parent. |
| 21A | 1.86 | 3.00 | This response acknowledges the client's feelings of isolation and validates their emotional state. It shows empathy and understanding, which can help the client feel heard and supported. | 2.33 | This response is appropriate. The helper reflects the underlying emotion of isolation that the person at-risk is expressing. By doing so, they validate the person's feelings without directly agreeing with or challenging their statement. This empathetic response can help the person feel understood and may encourage them to open up more about their feelings and experiences. | 2.00 | Helper A validates the client's feelings of isolation without judgment. It opens the door for further discussion about the client's relationships and support system. |
| 21B | -1.57 | 0.67 | This response invites the client to explore the reasons behind their feelings, which can be helpful. However, it might come across as slightly challenging or probing rather than supportive. It’s still constructive but less empathetic than the first response. | 1.00 | This response is marginally appropriate. While it seeks to explore the person's thoughts further, which can be beneficial, it might come across as slightly challenging or dismissive of their current feelings. The use of "anymore" also assumes a change in the person's perception, which may not be accurate. However, this response does show an interest in understanding the person's perspective, which can be helpful in building rapport and gaining more insight into their situation. | -0.33 | While asking "why" can be helpful at times, here it might put the burden on the client to justify their feelings. It's more helpful to first acknowledge the client's emotional state before delving into the reasons behind it. |
| 22A | -2.71 | -2.00 | This response is overly optimistic and dismissive of the person's feelings of hopelessness. It minimizes their experience and can come across as invalidating, potentially making the person feel worse. | -2.00 | This response is inappropriate. It dismisses the person's feelings of hopelessness and frustration with a forced positivity that doesn't acknowledge their experience. The helper's attempt to push for a "bright side" and insistence that there "must be" something to make things better can come across as dismissive and may make the person feel misunderstood or that their feelings are invalid. This approach could potentially shut down further communication. | -1.33 | While Helper A technically addresses the concern, it feels impersonal and robotic. It focuses on the helper's job rather than building a genuine connection with the client. |
| 22B | 1.43 | 2.33 | This response acknowledges the client's feelings of hopelessness and past experiences with therapy. It also gently prompts the client to consider other sources of support they may have found helpful, showing empathy and a willingness to explore alternative solutions. | 2.33 | This response is appropriate. The helper first acknowledges and validates the person's feelings of hopelessness, which shows empathy and understanding. Then, they gently explore other potential sources of support without dismissing the person's negative experience with therapy. This approach respects the person's feelings while also subtly introducing the idea that help might still be possible, potentially opening up a conversation about alternative sources of support. | 2.33 | It validates the client's feeling of hopelessness by acknowledging it. It gently explores alternative sources of support the client might have experienced. It offers a path forward by focusing on past positive experiences of support. |
| 23A | 1.57 | 3.00 | This response focuses on understanding the client's perspective and feelings about their diagnosis. It encourages the client to express their thoughts and emotions, fostering a deeper conversation. | 3.00 | This response is highly appropriate. Instead of focusing on the diagnosis or offering their own opinion, the helper redirects the conversation to the person's own understanding and feelings about their situation. This approach respects the person's perspective, encourages self-reflection, and allows for a deeper exploration of their current experiences and concerns. It also avoids the pitfall of contradicting or reinforcing a medical diagnosis, which would be outside the helper's role. | 2.67 | Helper A focuses on the client's understanding and feelings about the diagnosis. This allows the therapist to explore the client's perspective and build a collaborative approach to treatment. |
| 23B | -2.57 | -2.00 | This response questions the psychiatrist's diagnosis and suggests additional testing, which could undermine the client's trust in their current healthcare provider. It may cause confusion and does not directly address the client's feelings about the diagnosis. | -2.00 | This response is inappropriate. The helper is stepping outside their role by questioning a medical professional's diagnosis without having the necessary qualifications or information. Suggesting additional testing might increase the person's anxiety and confusion. This response doesn't address the person's feelings or experiences, and it could potentially undermine their trust in their current treatment plan. It also misses an opportunity to explore how the person is coping with their diagnosis and current situation. | -1.00 | Helper B undermines the client's trust in their psychiatrist and introduces doubt about the diagnosis. It's important for therapists to avoid questioning a diagnosis from another qualified professional, especially in front of the client. They can collaborate with the psychiatrist if necessary. |
| 24A | -2.43 | -2.00 | This response dismisses the client's feelings by outrightly denying their perception of their situation. It may come across as invalidating and unsupportive, potentially worsening the client's feelings of isolation. | -2.00 | This response is inappropriate. It directly contradicts the person's feelings and experiences, which can make them feel invalidated and misunderstood. The helper's assertion that "lots of people" care about them, without knowing the details of their situation, could come across as dismissive or insensitive. This approach might shut down further communication and reinforce the person's belief that no one understands their situation. | -1.00 | Helper A dismisses the client's feelings of isolation and distrust. While true that there might be supportive people, it's important to acknowledge the client's current per |
| 24B | 2.14 | 3.00 | This response acknowledges the client's difficulty in finding help and validates their feelings of mistrust. It shows empathy and understanding, which can help the client feel more supported. | 3.00 | This response is highly appropriate. The helper acknowledges and validates the person's feelings of isolation and distrust without agreeing or disagreeing with their statement. By empathizing with the difficulty of their situation, the helper shows understanding and creates a safe space for further discussion. This approach can help build trust and may encourage the person to open up more about their experiences and feelings. | 2.00 | Helper B validates the client's difficulty in seeking help due to their lack of trust. This shows empathy and understands the client's perspective. |
| 25A | -2.57 | -2.33 | This response suggests that the client get some sleep and call back later, which might not be appropriate if the client's slurred speech is due to substance use or an immediate crisis. It risks missing an opportunity to provide urgent help. | -3.00 | This response is highly inappropriate. Given that the person's voice is slurred and unclear, there's a possibility of substance use or a medical emergency. Suggesting they "get some sleep" and call back later could be dangerous if the person is in a crisis situation. This response fails to address the potential urgency of the situation and could leave the person at risk. | 2.00 | Helper A identifies the client's state of fatigue and offers a reasonable solution (calling back when rested) that allows for further assessment in the morning. |
| 25B | 2.43 | 2.67 | This response directly addresses the client's current state and seeks to understand if they have taken anything that might be affecting their speech. It shows concern for the client's immediate safety and opens the conversation to understand the situation better, which is crucial in assessing risk and providing appropriate support. | 2.67 | This response is highly appropriate. The helper immediately recognizes that something is wrong based on the person's voice and asks a critical question to assess the situation. By asking if the person has taken anything, the helper is trying to determine if there's a potential overdose or substance-related emergency. This question is crucial for understanding the level of risk and deciding on the appropriate next steps, which may include contacting emergency services if necessary. | -0.33 | While Helper B acknowledges the slurred speech, directly asking about substance use can be confrontational at this stage. It's important to prioritize safety and build rapport before potentially sensitive topics are explored. Helper A's approach allows for further conversation in the morning where substance use could be indirectly addressed. |
